# Association Between Dietary Patterns and Plasma Lipid Biomarker and Female Breast Cancer Risk: Comparison of Latent Class Analysis (LCA) and Factor Analysis (FA)

**DOI:** 10.3389/fnut.2021.645398

**Published:** 2021-12-09

**Authors:** Shang Cao, Linchen Liu, Qianrang Zhu, Zheng Zhu, Jinyi Zhou, Pingmin Wei, Ming Wu

**Affiliations:** ^1^Department of Epidemiology and Health Statistics, Southeast University, Nanjing, China; ^2^Department of Rheumatology, School of Medicine, Zhongda Hospital, Southeast University, Nanjing, China; ^3^Department of Chronic Disease Control, Jiangsu Provincial Center for Disease Control and Prevention, Nanjing, China

**Keywords:** dietary patterns, latent class analysis (LCA), factor analysis (FA), plasma lipid biomarkers, breast cancer

## Abstract

**Background:** Diet research focuses on the characteristics of “dietary patterns” regardless of the statistical methods used to derive them. However, the solutions to these methods are both conceptually and statistically different.

**Methods:** We compared factor analysis (FA) and latent class analysis (LCA) methods to identify the dietary patterns of participants in the Chinese Wuxi Exposure and Breast Cancer Study, a population-based case-control study that included 818 patients and 935 healthy controls. We examined the association between dietary patterns and plasma lipid markers and the breast cancer risk.

**Results:** Factor analysis grouped correlated food items into five factors, while LCA classified the subjects into four mutually exclusive classes. For FA, we found that the *Prudent*-factor was associated with a lower risk of breast cancer [4th vs. 1st quartile: odds ratio (OR) for 0.70, 95% CI = 0.52, 0.95], whereas the *Picky*-factor was associated with a higher risk (4th vs. 1st quartile: OR for 1.35, 95% CI = 1.00, 1.81). For LCA, using the *Prudent*-class as the reference, the *Picky*-class has a positive association with the risk of breast cancer (OR for 1.42, 95% CI = 1.06, 1.90). The multivariate-adjusted model containing all of the factors was better than that containing all of the classes in predicting HDL cholesterol (*p* = 0.04), triacylglycerols (*p* = 0.03), blood glucose (*p* = 0.04), apolipoprotein A1 (*p* = 0.02), and high-sensitivity C-reactive protein (*p* = 0.02), but was weaker than that in predicting the breast cancer risk (*p* = 0.03).

**Conclusion:** Factor analysis is useful for understanding which foods are consumed in combination and for studying the associations with biomarkers, while LCA is useful for classifying individuals into mutually exclusive subgroups and compares the disease risk between the groups.

## Introduction

The interest in dietary patterns is well-founded in nutritional epidemiology, in light of the limitation of the traditional single-nutrient approach (1–6). Dietary patterns can integrate complex interactions of diet exposures and bypass problems generated due to multiple testing and a high correlation among these exposures (1, 7). Due to the presence of dietary patterns, a relationship between diet and health outcomes is simplified and robust (2, 8, 9).

Generally, two main ideas are used to derive dietary patterns, *a priori* methods by using a predefined dietary pattern and fitting the data into the indices, namely the diet quality index (DQI) (10–12), or posterior methods by data-driven reduction techniques to explore dietary patterns, namely factor analysis (FA), principal component analysis (PCA), and cluster analysis (CA) (12, 13). The dietary patterns derived from “*a priori*” method have a clear explanation in the biological sense, while the “posterior” methods can obtain more information.

**Figure 1:**
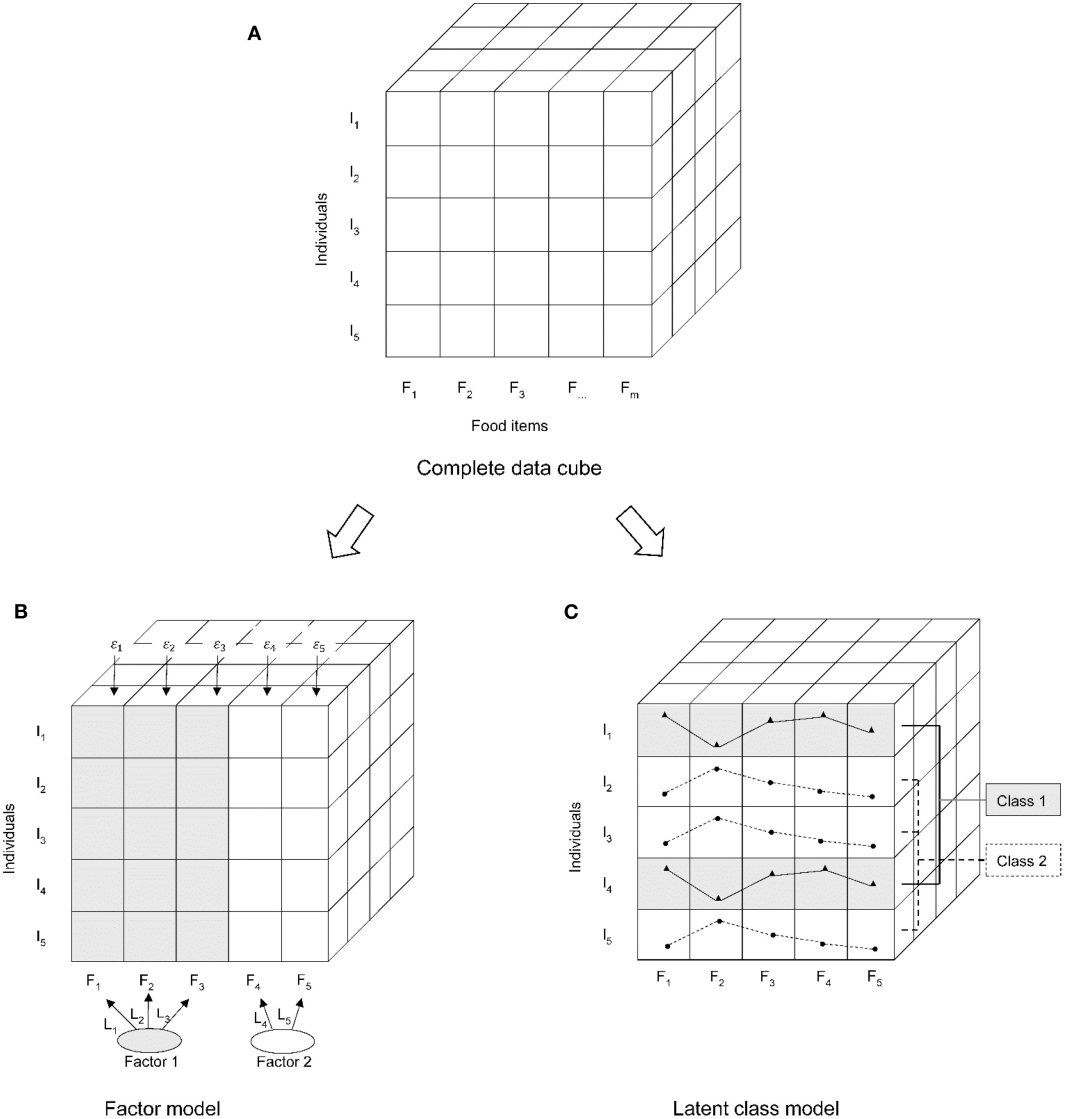
Differences in technical processing between the latent class analysis (LCA) and factor analysis (FA). **(A)** Data structure; **(B)** FA is a variable-oriented data reduction technique; **(C)** LCA is a person-centered classification technique. I, individuals; F, food items.

In the “posterior” methods, FA simplifies the diet data into dietary patterns based on the correlation between foods. It postulates that the created statistical model can explain this correlation through a limited number of underlying factors, and give factor scores to individuals for all the derived factors (13, 14). PCA and FA are closely related, the main difference is that FA assumes a certain statistical model for the existing data sets, while PCA does not rely on statistical assumptions and is mainly a mathematical method (15). CA simplifies the diet data into dietary patterns based on the differences of individuals in the mean dietary intake, and each individual belongs to only one cluster (13, 16). Recently, a novel CA method, latent class analysis (LCA) originating from psychology (17, 18), has been used in nutritional epidemiology (19, 20). LCA is similar to a non-hierarchical clustering analysis, but LCA is a model-based clustering method not a partition optimized based on numerical criteria (21). Because LCA relaxes the strict assumptions on conditional independence and the same error variance of all outcomes in clustering, it shows a better model fit (19). The main difference in concepts between FA and LCA is based on “person-centered” or “variable-oriented” [(22); Figure 1]. FA explains the correlations between many observed variables through few underlying continuous latent variables. LCA classifies participants into mutually exclusive groups, rather than a joint classification of the factors (23).

However, most diet studies focus on the characteristics of “dietary patterns,” such as the “*Western*” or “*Prudent*” dietary pattern, and regardless of what statistical methods are used to derive them. The effects are combined based only on the term of dietary pattern in some meta-analyses studies (24, 25). In fact, these approaches are both conceptually and technically different (4). When applied indiscriminately to the studies of associations with health outcomes, it may affect the reliability and generality of the results. In addition, the relationship between dietary effects, plasma lipids, and the breast cancer risk is complex, plasma lipids and lipoprotein are influenced by weight and diet and may be related to breast cancer risk factors. For example, the higher mammography density is considered to be a strong risk factor for breast cancer (26), which is related to increased levels of HDL-C and decreased levels of LDL-C (27). Some prospective clinical research suggested that high levels of TC and HDL-C increased the incidence of breast cancer (28–30). However, the conclusion is not consistent. A recent meta-analysis of the association between blood lipid levels and female breast cancer implicated no significant differences in the levels of total cholesterol, low-density lipoprotein cholesterol between cases and controls (31). Therefore, a direct comparison of methods of deriving dietary patterns is necessary, which would be useful to unravel the obscured relationship between diet, lipid profile levels, and the disease status and in moving the field forward. This study aimed to compare the dietary patterns derived from LCA and FA methods and their relation to plasma lipid biomarkers and female breast cancer risk.

## Methods

### Study Design and Subjects

Subjects came from a population-based case-control study involving biology, diet, lifestyle, and environmental factors impact on the risk of breast cancer in Asian women. All subjects were adult women and restricted to local residents who have lived in Wuxi for at least 5 years. All newly diagnosed female breast cancers (ICD code: C50) among local residents identified by cancer registries are eligible to be included as cases. Secondary and recurrent cancers will be excluded. Controls were derived from the local area as cases and will be 1:1 individually matched with cases by age (±2 years) and residence. As personal information such as name, address, date of birth, and sex for all residents is available in the local demographic information database, eligible controls are randomly identified from this database. For choosing each control, two additional subjects will be selected as a backup at the same time. When the first control could not be interviewed, an alternative will be enrolled in the study. The selection procedure will be repeated until an eligible subject is interviewed. A total of 1,042 eligible breast cancer cases and 1,042 health controls were identified during the study period. About 818 cases and 935 controls agreed to participate, with a frequency match (cases and controls have the same distributions over age and residence). We excluded 77 cases and 75 controls because of extreme values in total calorie intake (<500 or >5,000 kcal) and 46 cases and 56 controls missing the information on adjusting covariant variables. A total of 695 cases and 804 controls were finally included in this study. This study was conducted according to the guidelines laid down in the Declaration of Helsinki, and all procedures involving human subjects/patients were approved by the Jiangsu Center for Disease Control and Prevention ethical committee. Written informed consent was obtained from all subjects/patients.

### Plasma Lipid Measurements

In the blood samples of all subjects, a series of plasma lipid biomarkers, including LDL cholesterol, HDL cholesterol, total cholesterol, triacylglycerols, blood glucose, apolipoprotein A1, apolipoprotein B, and high-sensitivity C-reactive protein, were measured. Anantecubital venous blood sample was drawn from the study subjects after they had fasted overnight. Blood glucose, concentrations of triacylglycerols, and total cholesterol were measured by using an enzymatic method (GPO-POD method and GHOD-POD method), HDL cholesterol and LDL cholesterol were measured by a homogeneous enzymatic method, apolipoprotein A1, apolipoprotein B, and high-sensitivity C-reactive protein were measured by an immunoturbidimetric method, and all plasma lipid measurements were done using the Roche Chemistry Analyzer (cobas c701).

### Dietary Assessment

The diet was measured by a validated, semi-quantitative food frequency questionnaire (FFQ), which included 149 food items. The 149 food items can be further classified into 18 predefined food groups based on similarities in nutrient profile and culinary usage. A detailed description and reliability verification of the FFQ can be found in the previously published study (32). Total energy intake is based on the Chinese Food Composition Database (2018, 6th version).

### Dietary Pattern Analysis

*Latent class analysis*: LCA for dietary pattern derivation is described briefly as follows:

Latent class analysis is a conditional Gaussian finite mixture model [FMM; (19)]. The identification of dietary patterns can be considered as there are subgroups who are distinguished by their dietary profiles in the population and have different food consumption probability distributions. FMM is particularly suited to the problem of identifying the subgroups that are defined in this manner. In FMM, the overall population probability density is expressed as a finite sum of well-defined component densities, with each density representing a subgroup.

An FMM can be written as


(1) $$\begin{array}{r} f\left({\text{y}}_{i}|\theta \right)\;=\;\sum_{k=1}^{K}\pi_{k}f_{k}\left({\text{y}}_{i}|\theta_{k}\right) \end{array}$$

In Equation (1), *y*_*i*_ is a vector of observations on *J* feature variables for the *i*th subject, *K* is the chosen number of subgroups, π_*k*_ is the probability of subgroup membership (or mixing proportion) which sums to 1 over subgroups and θ is the set of model parameters that are to be estimated. If the feature variables are continuous, it is usually assumed that the *K* probability densities *f*_1_,…,*f*_*k*_ are multivariate normal. The most general solution involves estimating a separate set of means, variances, and covariances for each component density, as well as the mixing proportions.

The details please refer to our previous study (33). The dietary classes derived from LCA adjusted the energy intake of each subject and were interpreted and named according to the conditional probabilities of food group intake, using controls only. The number of classes was determined by the Bayesian Information Criterion (BIC), Lo–Mendel–Rubin likelihood ratio (LMR) test, and entropy value (34, 35). The dietary classes were derived from LCA.

Factor analysis: FA is the most commonly used method to derive dietary patterns, briefly described as follows.

The identification of dietary patterns in FA can be regarded as a problem of few latent variables to explain the correlation between many observed variables, which is achieved by dividing a covariance between the observed variables. These continuous explanatory latent variables are called “factors.”

Assuming that the intake of *n* subjects in *P* dietary variables *X*_1_, *X*_2_, …, *X*_*P*_ is measured, where *i* variables can be written as a linear combination based on *m* factors *F*_1_, *F*_2_, …, *F*_*m*_. When *m*< *p*, a FA can be expressed in Equation (2) as


(2) $$\begin{array}{r} X_{i}=a_{i1}F_{1}+a_{i2}F_{2}+\ldots +a_{im}F_{m}+e_{i} \end{array}$$

*a*_*is*_ is the factor loading of the variable *i*, and *e*_*i*_ is the part of the variable *X*_*i*_ that cannot be “explained” by the factors.

We first performed an exploratory factor analysis (EFA) on 18 food groups using weighted least squares and derived the factors by orthogonal Varimax rotation. The number of factors left is based on the characteristic root and the variance interpretation. Next, we constructed a confirmatory factor analysis (CFA) model that only included food groups with the loading value ≥ 0.25 in EFA, allowing food groups to load on multiple factors. Both EFA and CFA analyses use controls only and adjust each subject's energy intake.

### Statistical Analysis

To compare the characteristics of the dietary patterns derived from LCA and FA, we calculated consumption conditional probabilities and factor loadings for each food group and compared factor scores' means (±SD) for each class.

To compare the association between the dietary patterns derived by LCA or FA and plasma lipid biomarkers, we used a multivariate-adjusted linear regression to examine individual associations between each class or each factor with each plasma lipid biomarker. Indicator variables (aka, dummy variables) were created for each class, while the factors remained as continuous variables (*z-*scores). A separate linear regression model was constructed for each individual class or factor for each plasma lipid biomarker (plasma lipid biomarker as an outcome variable). Each dietary pattern (derived by LCA or FA) will be tested in eight separate regression models to examine the associations between a dietary pattern and LDL cholesterol, HDL cholesterol, total cholesterol, triacylglycerols, blood glucose, apolipoprotein A1, apolipoprotein B, and high-sensitivity C-reactive protein, respectively. The multi-regression analysis of each dietary pattern derived by FA or LCA will be performed two times. We will first adjust age (age at diagnosis for cases or enrollment for controls, by years) and BMI (kg/m^2^) and further adjust area (urban and rural), education (ordered as illiterate and primary, middle, and high school, University and above), smoking (no or yes: including smoking and second-hand smoking ≥ 3 day/week), moderate physical activity (min/day), oral contraceptive use (no or yes: current use or ever use), hormone replacement therapy (no or yes: current use or ever use), age at menarche (by years), age at first full-term delivery (by years), parity (ordered as 0, 1, 2, or ≥3), family history of breast cancer (no or yes: in a first-degree relative), history of benign breast disease (no or yes: including lactation mastitis, plasma cell mastitis, cyclomastopathy, fibroadenoma of breast, and galactocele), breastfeeding (no or yes), height (in cm), energy intake (kcal/extra-administrative) and menopausal status (premenopausal, postmenopausal, postmenopausal as the absence of menstruation in the past 12 months). To further compare dietary patterns in relation to health outcomes (included plasma lipid biomarkers and breast cancer risk), we built a linear regression model that included all the factors and another linear regression model that included all the classes and then compared them using *Pitman's* test to see which solution better predicted the outcomes.

To examine the association between dietary patterns and the disease risk, we calculated standardized factor scores and Bayesian posterior probability for each subject, so that all the subjects were assigned with a score for each dietary pattern, and all the subjects were assigned with a latent class, based on their FFQ intake. The logistic regression models were used to estimate the odds ratio (OR) and their 95% CIs. For FA, because the factors are not mutually exclusive and the factor scores are continuous variables, we divided the factor score of each dietary pattern into quartiles and examined their association with the breast cancer risk, with a reference of the lowest quartile. For LCA, because the classes are mutually exclusive, we estimate the risk of breast cancer directly for mutually exclusive classes compared with a reference class.

Latent class analysis and FA were conducted using MPLUS (V8.3; Muthén & Muthén, Los Angeles, CA, USA) (36), and other statistical analyses were conducted using R version 4.0.2 (The R Project for Statistical Computing, USA; https://www.r-project.org/).

## Results

### Dietary Derived by LCA

The dietary patterns derived from LCA were described in our previous studies (33). As described briefly below, latent class models were fitted for two to six classes, and the four classes were chosen. The food consumption conditional probability from the selected food groups for the four classes was presented in Table 1. We named the classes as follows: *Prudent, Chinese traditional* (short for *Chinese* below)*, Western*, and *Picky*. The *Prudent* class was characterized by a high probability of consuming healthy foods like cereals, aquatic products, fruits, vegetables, soy foods, and nuts. Compared with the other three classes, women in the *Picky*-class were characterized by higher extreme probabilities of non-consumption of specific foods.

**Table 1:** Food consumption level conditional probabilities of dietary pattern classes, latent class analysis (LCA)^a^^,^ ^b^.

	**Class 1:**		**Class 2:**		**Class 3:**		**Class 4:**	
	***Prudent***		***Western***		***Chinese***		***Picky***	
**Food group**	**High**	**No**	**High**	**No**	**High**	**No**	**High**	**No**
	**consumption**	**consumption**	**consumption**	**consumption**	**consumption**	**consumption**	**consumption**	**consumption**
Rice/Flour	0.08	0.25	0.16	0.40	0.10	0.26	0.24	0.17
Cereals	0.35	0.24	0.28	0.15	0.25	0.12	0.17	0.56
Fried food	0.13	0.24	0.46	0.12	0.00	0.50	0.02	0.73
Meat	0.47	0.03	0.79	0.01	0.03	0.00	0.15	0.10
Poultry	0.46	0.26	0.71	0.00	0.29	0.05	0.23	0.36
Aquatics	0.56	0.02	0.55	0.00	0.29	0.01	0.27	0.13
Eggs	0.05	0.25	0.22	0.01	0.06	0.03	0.07	0.36
Milk	0.01	0.47	0.04	0.26	0.01	0.38	0.01	0.85
Fruits	0.25	0.04	0.18	0.00	0.30	0.01	0.14	0.32
Vegetables	0.26	0.01	0.27	0.00	0.17	0.00	0.37	0.04
Soy foods	0.40	0.17	0.49	0.09	0.25	0.06	0.18	0.38
Nuts	0.26	0.34	0.25	0.08	0.14	0.15	0.11	0.67
Cakes	0.23	0.51	0.33	0.15	0.13	0.31	0.10	0.76
SSB	0.02	0.98	0.25	0.75	0.11	0.89	0.05	0.95
Fresh juice	0.06	0.94	0.27	0.73	0.11	0.89	0.02	0.98
Soft drink	0.06	0.94	0.47	0.53	0.18	0.82	0.07	0.93
Pickled foods	0.12	0.44	0.25	0.19	0.16	0.32	0.24	0.33
Coffee	0.08	0.92	0.26	0.74	0.08	0.92	0.02	0.99

a *Classes were derived using LCA on 18 food groups based on 804 controls*.

b *Conditional probabilities of food group consumption were categorized into four levels: tertiles of non-zero consumption and no consumption (calculated from controls). Because there were <20% of women consumed sugar strengthened beverage (SSB), fresh juice, soft drink, or coffee, we set the consumption of these foods as binary variables (consumed or no). While rice/flour was consumed almost ubiquitously, there were only tertiles of consumption and no non-consumption category*.

### Dietary Derived by FA

According to the scree plot and characteristic root from EFA (the first six eigen values were 2.57, 1.66, 1.44, 1.29, 1.18, and 1.01), we extracted five factors, which explain ~45.21% of the total variance. Factor 1 with a high factor loading in cereals, aquatics, milk, fruits, soy foods, nuts, cakes, and fresh juice, named as *Prudent*-factor; Factor 2 with a high factor loading in cakes, sugar strengthened beverage (SSB), fresh juice, soft drinks, pickled foods, and coffee, named as *Sugar*-factor. Factor 3 with a high factor loading in fried foods and red meat, named as *Western*-factor; Factor 4 with a high factor loading in poultry, eggs, and soy foods, named as *Chinese traditional*-factor (short for *Chinese*); Factor 5 with a high factor loading in vegetables, soy foods, and pickled foods, named as *Picky*-factor.

The CFA model only included food groups with loading ≥0.25 in EFA. The factor loadings from EFA and CFA were almost similar except for coffee for *Picky*-factor and fresh juice for *Sugar*-factor (Table 2). Therefore, we kept the names given from EFA for the dietary patterns assessed by CFA. After excluding food groups with the factor loading <0.25, the model was more concise and the goodness of fit did not decrease (results not shown). We examined the overall correlations among the five factors and found a significant difference (*p* < 0.001) compared to the hypothesis of being zero (for details see Supplementary Figure 1).

**Table 2:** Selected exploratory and confirmatory factor loadings for the five-factor model, factor analysis (FA)^a^.

	**EFA**					**CFA^b^**				
**Food group**	**Factor 1:**	**Factor 2:**	**Factor 3:**	**Factor 4:**	**Factor 5:**	**Factor 1:**	**Factor 2:**	**Factor 3:**	**Factor 4:**	**Factor 5:**
	***Prudent***	***Sugar***	***Western***	***Chinese***	***Picky***	***Prudent***	***Sugar***	***Western***	***Chinese***	***Picky***
Rice/Flour	−0.12	−0.02	−0.02	0.03	0.46	–	–	–	–	0.54
Cereals	0.45	−0.11	−0.06	0.07	0.07	0.35	–	–	–	–
Fried food	0.10	0.17	0.78	0.03	0.01	–	–	0.78	–	–
Meat	0.03	0.04	0.88	0.24	0.05	–	–	0.89	–	–
Poultry	0.08	0.04	0.10	0.92	−0.18	–	–	–	0.61	–
Aquatics	0.24	0.01	0.10	0.13	−0.01	0.30	–	–	–
Eggs	0.20	0.15	0.12	0.41	0.08		–	–	0.64	–
Milk	0.48	0.10	0.03	0.01	−0.23	0.49	–	–	–	–
Fruits	0.48	−0.08	−0.02	0.07	−0.16	0.43	–	–	–	–
Vegetables	0.02	0.01	0.04	−0.07	0.41		–	–	–	0.35
Soy foods	0.32	0.13	0.09	0.26	0.25	0.33	–	–	0.29	0.30
Nuts	0.50	0.15	0.11	0.05	0.08	0.55	–	–	–	–
Cakes	0.42	0.32	0.14	0.01	−0.02	0.43	0.25	–	–	–
SSB	0.04	0.76	0.08	0.01	0.06		0.72	–	–	–
Fresh juice	0.48	0.31	−0.04	0.01	−0.20	0.56	–	–	–
Soft drink	0.11	0.79	0.04	0.14	−0.04	–	0.86	–	–	–
Pickled foods	−0.09	0.34	0.04	0.12	0.24	–	0.34	–	–	0.27
Coffee	0.33	0.45	0.17	0.01	−0.26	0.37	0.36	–	–	–

a *Factors were derived using FA on 18 food groups based on 804 controls*.

b *Food groups with factor loading <0.25 are excluded for simplicity*.

### Comparison Between LCA and FA

**Figure 2:**
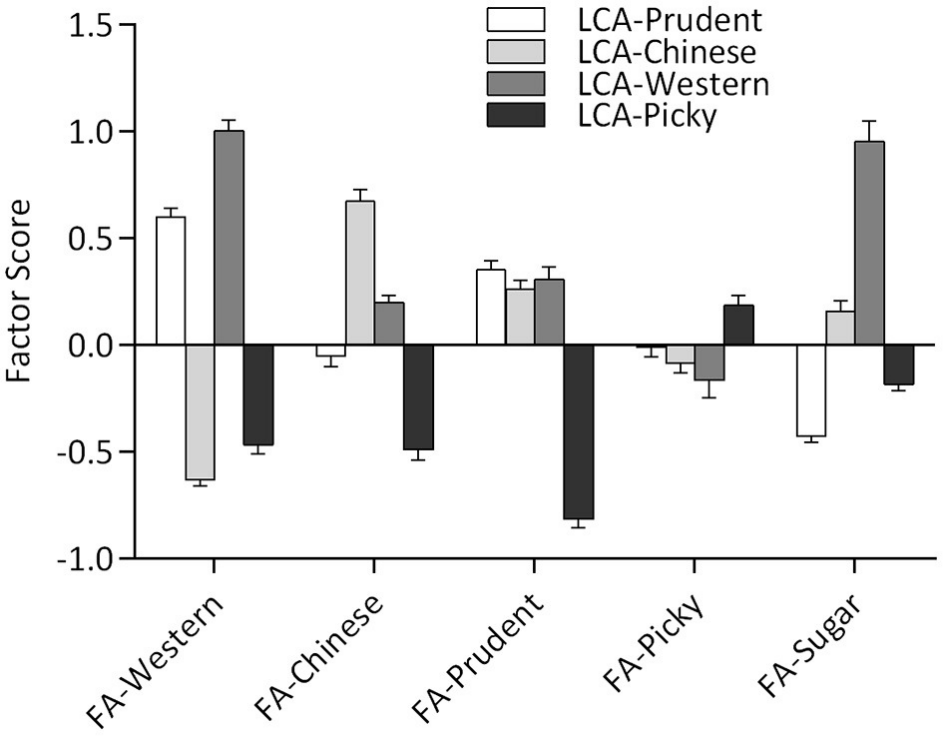
Factor scores' means by latent class, four classes on five factor scores.

Latent class analysis and FA methods identified similar dietary patterns based on the same data sets, which have similar diet characteristics from the conditional probabilities of LCA and factor loadings of FA (Tables 1, 2). Latent classes derived from LCA have higher factor scores on corresponding latent factors, as shown in Figure 2. Besides, the *Western*-class also had the highest factor score for *Sugar*-factor. The *Picky*-class had the lowest factor score for *Prudent*-factor and also had the factor score less than zero for *Western*-factor, *Chinese*-factor, *Prudent*-factor, and *Sugar*-factor. Although the *Prudent*-class had higher means for the *Prudent*-factor score, the factor score between the *Chinese*-class and *Western*-class was not significantly different (results not shown).

### Dietary Patterns and Plasma Lipid Biomarkers

In the multivariate-adjusted regression models for the classes derived by LCA, individuals in the *Western*-class had higher total cholesterol (β = 0.23; *p* < 0.01), triacylglycerols (β = 0.28; *p* < 0.01), blood glucose (β = 0.29; *p* < 0.01), and apolipoprotein B (β = 0.08; *p* < 0.01) than those who are not in the *Western*-class. Individuals in the *Picky*-class had higher triacylglycerols (β = 0.23; *p* < 0.01) and blood glucose (β = 0.29; *p* < 0.01) than those who are not in the *Picky*-class (Table 3).

**Table 3:** Association between dietary patterns (classes) and plasma lipid biomarkers, regression coefficients (β)^a^.

**Dietary pattern^e^**	**LDL cholesterol**	**HDL cholesterol**	**Total cholesterol**	**Triacylglycerols**	**Blood glucose**	**Apolipoprotein A1**	**Apolipoprotein B**	**High-sensitivity C-reactive Protein**
**Class 1:** ***Prudent***								
Adjusted for age and BMI	−0.01 (0.07)	0.02 (0.03)	−0.05 (0.08)	−0.07 (0.07)	−0.11 (0.10)	−0.00 (0.02)	−0.02 (0.02)	−0.03 (0.13)
Multivariate adjusted^d^	−0.01 (0.07)	0.02 (0.03)	−0.04 (0.08)	−0.08 (0.07)	−0.11 (0.10)	−0.00 (0.02)	−0.02 (0.02)	−0.04 (0.14)
**Class 2:** ***Western***								
Adjusted for age and BMI	0.15 (0.09)	−0.03 (0.04)	0.22 (0.10)^b^	0.27 (0.09)^c^	0.26 (0.13)^b^	0.03 (0.02)	0.07 (0.03)^c^	0.03 (0.17)
Multivariate adjusted^d^	0.17 (0.09)	−0.03 (0.04)	0.23 (0.10)^b^	0.28 (0.09)^c^	0.29 (0.13)^b^	0.03 (0.02)	0.08 (0.03)^c^	0.03 (0.17)
**Class 3:** ***Chinese***								
Adjusted for age and BMI	0.06 (0.07)	0.03 (0.03)	0.04 (0.07)	−0.10 (0.07)	−0.17 (0.10)	0.01 (0.02)	0.01 (0.02)	0.01 (0.13)
Multivariate adjusted^d^	0.06 (0.07)	0.03 (0.03)	0.04 (0.07)	−0.10 (0.07)	−0.16 (0.10)	0.02 (0.02)	0.01 (0.02)	0.01 (0.13)
**Class 4:** ***Picky***								
Adjusted for age and BMI	0.03 (0.08)	−0.04 (0.03)	0.06 (0.08)	0.23 (0.07)^c^	0.27 (0.11)^b^	0.00 (0.02)	0.02 (0.02)	0.05 (0.15)
Multivariate adjusted^d^	0.04 (0.08)	−0.03 (0.03)	0.07 (0.08)	0.23 (0.07)^c^	0.29 (0.11)^b^	0.00 (0.02)	0.02 (0.02)	0.06 (0.15)

a *SE in parentheses*.

b *p < 0.05*.

c *p < 0.01*.

d *Multivariate models were adjusted for age, BMI, area, education, smoking, age at menarche, age at first full–term delivery, parity, age at menopause, parity, family history of breast cancer, history of benign breast disease, use of HRT, use of oral contraceptives, breastfeeding, moderate physical activity, height, body mass index, total energy intake, and menopausal status*.

e *Association between dietary patterns (classes) and plasma lipid biomarkers based on 804 controls*.

In multivariate-adjusted regression models for the factors derived by FA, the *Prudent*-factor was inversely related to triacylglycerols (β = −0.12; *p* < 0.01), blood glucose (β = −0.13; *p* < 0.01), apolipoprotein B (β = −0.02; *p* < 0.01), and high-sensitivity C-reactive protein (β = −0.13; *p* < 0.01), whereas the *Picky*-factor was directly associated with triacylglycerols (β = 0.07; *p* < 0.05), apolipoprotein A1(β = 0.02; *p* < 0.05), and high-sensitivity C-reactive protein (β = 0.14; *p* < 0.05). Individuals in the *Sugar*-factor had higher LDL cholesterol (β = 0.09; *p* < 0.01), total cholesterol (β = 0.10; *p* < 0.01), triacylglycerols (β = 0.06; *p* < 0.01), blood glucose (β = 0.15; *p* < 0.01), and apolipoprotein B (β = 0.03; *p* < 0.01; Table 4). Because the factors are continuous variables (*z-*scores), β here means 1 mg/dl for a 1-unit increase in *z-*score.

**Table 4:** Association between dietary patterns (factors) and plasma lipid biomarkers, regression coefficients (β)^a^.

**Dietary pattern^e^**	**LDL cholesterol**	**HDL cholesterol**	**Total cholesterol**	**Triacylglycerols**	**Blood glucose**	**Apolipoprotein A1**	**Apolipoprotein B**	**High-sensitivity C-reactive Protein**
**Factor 1:** ***Prudent***								
Adjusted for age and BMI	−0.04 (0.03)	0.03 (0.01)^b^	−0.03 (0.03)	−0.12 (0.03)^c^	−0.13 (0.05)^c^	0.00 (0.01)	−0.02 (0.01)^b^	−0.13 (0.06)^b^
Multivariate adjusted^d^	−0.04 (0.03)	0.02 (0.01)	−0.03 (0.03)	−0.12 (0.03)^c^	−0.13 (0.05)^c^	0.00 (0.01)	−0.02 (0.01)^b^	−0.13 (0.06)^b^
**Factor 2:** ***Sugar***								
Adjusted for age and BMI	0.09 (0.03)^c^	−0.02 (0.01)	0.10 (0.03)^c^	0.06 (0.03)^b^	0.15 (0.05)^c^	0.01 (0.01)	0.03 (0.01)^c^	0.04 (0.06)
Multivariate adjusted^d^	0.09 (0.03)^c^	−0.02 (0.01)	0.10 (0.03)^c^	0.06 (0.03)^b^	0.15 (0.05)^c^	0.01 (0.01)	0.03 (0.01)^c^	0.02 (0.06)
**Factor 3:** ***Western***								
Adjusted for age and BMI	−0.03 (0.03)	−0.01 (0.01)	0.00 (0.03)	0.05 (0.05)	−0.01 (0.01)	−0.01 (0.01)	0.05 (0.06)	0.05 (0.06)
Multivariate adjusted^d^	−0.03 (0.03)	−0.01 (0.01)	−0.04 (0.03)	0.00 (0.03)	0.04 (0.05)	−0.01 (0.01)	−0.01 (0.01)	0.05 (0.06)
**Factor 4:** ***Chinese***								
Adjusted for age and BMI	0.03 (0.03)	0.02 (0.01)	0.03 (0.04)	−0.05 (0.03)	−0.00 (0.05)	0.02 (0.01)	0.01 (0.01)	−0.06 (0.06)
Multivariate adjusted^d^	0.02 (0.03)	0.02 (0.01)	0.02 (0.04)	−0.05 (0.03)	−0.01 (0.05)	0.02 (0.01)	0.00 (0.00)	−0.06 (0.06)
**Factor 5:** ***Picky***								
Adjusted for age and BMI	−0.01 (0.03)	0.01 (0.01)	0.02 (0.03)	0.07 (0.03)^b^	−0.01 (0.04)	0.02 (0.01)^b^	0.00 (0.01)	0.14 (0.06)^b^
Multivariate adjusted^d^	−0.01 (0.03)	0.01 (0.01)	0.02 (0.03)	0.07 (0.03)^b^	−0.01 (0.04)	0.02 (0.01)^b^	0.00 (0.01)	0.14 (0.06)^b^

a *SE in parentheses*.

b *p < 0.05*.

c *p < 0.01*.

d *Multivariate models were adjusted for age, BMI, area, education, smoking, age at menarche, age at first full-term delivery, parity, age at menopause, parity, family history of breast cancer, history of benign breast disease, use of HRT, use of oral contraceptives, breastfeeding, moderate physical activity, height, body mass index, total energy intake, and menopausal status*.

e *Association between dietary patterns (factors) and plasma lipid biomarkers based on 804 controls*.

From the *Pitman's* test results, we found that the model containing all of the factors was slightly better than the model containing all of the classes in predicting HDL cholesterol (*p* = 0.04), triacylglycerols (*p* = 0.03), blood glucose (*p* = 0.04), apolipoprotein A1 (*p* = 0.02), high-sensitivity C-reactive protein (*p* = 0.02), but was weaker than that in predicting the breast cancer risk (*p* = 0.03; Table 5).

**Table 5:** The proportion of variability explained (*R*^2^) by regression models containing all classes or all factors in predicting plasma lipid biomarkers and *Pitman's* test.

**Dietary pattern^a^**	**LDL cholesterol**	**HDL cholesterol**	**Total cholesterol**	**Triacylglycerols**	**Blood glucose**	**Apolipoprotein A1**	**Apolipoprotein B**	**High-sensitivity C-reactive Protein**	**Breast cancer**
**Model 1: all classes**									
Classes only^b^	0.005	0.003	0.007	0.023	0.014	0.002	0.011	0.000	0.005
Adjusted for age and BMI	0.014	0.005	0.018	0.023	0.017	0.005	0.016	0.001	0.009
Multivariate adjusted^c^	0.112	0.023	0.141	0.091	0.055	0.039	0.131	0.021	0.023
**Model 2: all factors**									
Factors only	0.014	0.011	0.021	0.069	0.024	0.012	0.020	0.015	0.008
Adjusted for age and BMI	0.024	0.012	0.024	0.034	0.027	0.015	0.026	0.017	0.011
Multivariate adjusted^c^	0.114	0.031	0.141	0.099	0.063	0.049	0.131	0.031	0.014
*P*-value for Pitman's test	0.42	0.04	1.00	0.03	0.04	0.02	1.00	0.02	0.03

a *Association between dietary patterns (classes) and plasma lipid biomarkers and the breast cancer risk based on all subjects (695 cases, 804 controls)*.

b *Because the classes are categorical variables, regression models contain only three classes because one class as the reference*.

c *Multivariate models were adjusted for age, BMI, area, education, smoking, age at menarche, age at first full-term delivery, parity, age at menopause, parity, family history of breast cancer, history of benign breast disease, use of HRT, use of oral contraceptives, breastfeeding, moderate physical activity, height, body mass index, total energy intake, and menopausal status*.

### Dietary Patterns and Health Outcomes

For FA, the *Prudent*-factor was associated with a lower breast cancer risk (4th vs. 1st quartile: OR for 0.70, 95% CI: 0.52–0.95, *p*-trend = 0.0029), while the *Picky*-factor was associated with a higher breast cancer risk (4th vs. 1st quartile: OR for 1.35, 95% CI: 1.00–1.81, *p*-trend = 0.1220; Table 6). For LCA, we found that the *Prudent*-class was similar to the Mediterranean pattern in terms of the correlation with food intake. Using the *Prudent*-class as the reference, we found that individuals belonging to the *Picky*-class have a significant higher breast cancer risk (OR for 1.42, 95% CI = 1.06, 1.90) (Table 6).

**Table 6:** Associations between the dietary patterns derived by FA and LCA and health outcome (breast cancer)^a^, adjusted OR and 95% CI^b^.

**Factor analysis**	**Factor 1:**	**Factor 2:**	**Factor 3:**	**Factor 4:**	**Factor 5:**
	***Prudent***	***Sugar***	***Western***	***Chinese***	***Picky***
Quartile 1	1.00 (reference)	1.00 (reference)	1.00 (reference)	1.00 (reference)	1.00 (reference)
Quartile 2	1.12 (0.83, 1.50)	1.03 (0.77, 1.39)	0.81 (0.60, 1.09)	1.15 (0.80, 1.66)	1.39 (1.03, 1.86)
Quartile 3	0.77 (0.57, 1.03)	1.05 (0.78, 1.42)	1.05 (0.78, 1.42)	0.94 (0.63, 1.40)	1.19 (0.88, 1.60)
Quartile 4	0.70 (0.52, 0.95)	1.06 (0.79, 1.43)	0.95 (0.70, 1.28)	0.71 (0.46, 1.09)	1.35 (1.00, 1.81)
*P* for trend	0.0029	0.6832	0.8270	0.0940	0.1220
**Latent class analysis**	**Class 1:**	**–**	**Class 2:**	**Class 3:**	**Class 4:**
	***Prudent***	**–**	***Western***	***Chinese***	***Picky***
	1.00 (reference)	–	0.76 (0.53, 1.09)	0.86 (0.65, 1.14)	1.42 (1.06, 1.90)

a *Association between dietary patterns (classes) and plasma lipid biomarkers and the breast cancer risk based on all subjects (695 cases, 804 controls)*.

b *Adjusted for or age, BMI, area, education, smoking, age at menarche, age at first full-term delivery, parity, age at menopause, parity, family history of breast cancer, history of benign breast disease, use of HRT, use of oral contraceptives, breastfeeding, moderate physical activity, height, body mass index, total energy intake, and menopausal status*.

## Discussion

Nutritional studies have historically been focusing on specific nutrients or foods in isolation and oversimplified the complexity of foods (3, 6). A high degree of intercorrelation among various nutrients and foods makes it difficult to attribute effects to a single independent component, and the interpretation and application of results were limited (1, 5). Now, in nutrition epidemiology, the concept of food synergy has been convinced that nutrients exist in a purposeful biological sense in food. The dietary patterns that inherently account for interactions among nutrients and estimate overall dietary effects may provide a more robust approach for determining associations between diet and health outcomes (8, 9).

Although various methods have been developed to derive dietary patterns, there are still many challenges in an accurate identification of dietary patterns (37). Different statistical methods use different concepts and techniques to reduce the complex multidimensional nutritional data down to meaningfully observed dietary patterns. For example, the most commonly used FA method is “variance-oriented,” which is achieved by partitioning variances among variables and explaining the correlations between many observed variables through few underlying continuous latent variables. In contrast, LCA is a “person-oriented” approach, which models the distinct configurations of heterogeneity within a sample and divides the sample into mutually exclusive subgroups with different dietary structures (38, 39). When applying the dietary patterns derived from different methods indiscriminately to studies, it may affect the reliability and generalizability of the results.

The results of this study show that the dietary patterns derived from the different methods are both formally and biologically different. The FA approach summarizes five factors (“*Prudent*,” “*Western*,” “*Chinese traditional*,” “*Picky*,” and “*Sugar*”) based on the correlation of food group intake, LCA approach derives four classes (“*Prudent*,” “*Western*,” “*Chinese traditional*,” and “*Picky*”) based on the differences in a dietary structure of the study population. Despite on the basis of characteristics of the conditional probability of LCA and factor loading of FA as well as the factor scores of the latent class on the corresponding factors, the same-named dietary patterns are similar in diet characteristics. However, the FA method identified a typical food combination from a strong preference for sweet foods, while the LCA method did not derive the “pure” *Sugar*-class. On another side, the characteristics of the *Picky* pattern were high extreme probabilities of non-consumption on specific foods, which was only reflected in the LCA result.

**Figure 3:**
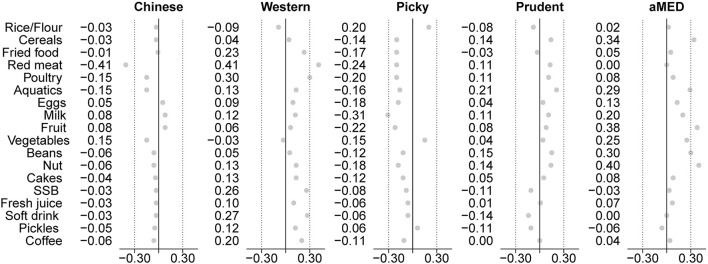
Correlations between food consumption and the dietary pattern, based on the posterior LCA method and the prior diet quality index (DQI) method. Compared with the data-driven “posterior” method, the “*a priori*” method has a clearer biological meaning under a certain diet pattern. This study found that in terms of its relevance to the specific food group, the *Prudent*-dietary pattern from LCA is similar to the Mediterranean dietary pattern.

Through examining the associations between dietary patterns and plasma lipid biomarkers, we found that the *Prudent*-dietary pattern characteristic of cereal, aquatics, fruits, soy foods, and nuts in case of its derivation by LCA or FA was inversely associated with triacylglycerols, blood glucose, and apolipoprotein B. While the *Picky* pattern was associated with triacylglycerols and blood glucose when derived by LCA and was associated with triacylglycerols, apolipoprotein A1, and high-sensitivity C-reactive protein when derived by FA. *Chinese traditional* and *Western* patterns were not significantly associated with any of the plasma lipid biomarkers regardless of using the LCA or FA method. Although the coefficients of pattern-plasma lipid biomarker regression from LCA and FA cannot be compared directly because the dietary patterns (classes) derived by LCA were treated as indicator variables and are dichotomous, whereas the dietary patterns (factors) derived by FA were treated as continuous variables (*z-*scores), the associations between dietary patterns and biomarkers were in a similar direction for both LCA and FA methods. When we compared a model containing all the classes with a model containing all the factors, we found that FA is slightly better than LCA in predicting some plasma lipid biomarkers (HDL cholesterol, triacylglycerols, blood glucose, apolipoprotein A1, and high-sensitivity C-reactive protein), while LCA is better than FA in predicting the breast cancer risk. Furthermore, we examined the dietary patterns-health outcome associations. Because the factors derived by FA are not mutually exclusive, an individual's dietary pattern can only be inferred by her factor score of the derived factors (40). We found that women with the highest quartile score of the *Prudent*-factor decreased 30% risk compared to women with the lowest quartile, and with robust linearity (*p*-trend = 0.0029). While women who follow a *Picky*-factor increase 35% risk of breast cancer, but there is insufficient evidence for considerable linearity (*p*-trend = 0.1220). In contrast, LCA classifies participants into mutually exclusive groups, the disease risk can be directly compared between groups, but need to select a reference first. We used the *Prudent*-class as the reference, which was similar to the recognized healthy dietary pattern (Mediterranean diet, Figure 3) and found that individuals belonging to the *Picky*-class have a 42% higher risk of breast cancer than those belonging to the *Prudent*-class.

The difference between the dietary patterns derived from LCA and FA methods can be explained by their concept and technology. FA summaries dietary patterns based on the correlation between foods intake. The methodological characteristics of FA may explain why the dietary patterns derived by FA are more closely related to plasma lipid biomarkers than those derived by LCA, and the synergy produced by highly correlated foods strengthens the relationship between dietary patterns and plasma lipid biomarkers (Figure 1). However, we cannot make a direct comparison of the risk of disease between individuals using the FA approach (40), which needs mutually exclusive subgroups and a chosen reference group. The challenge is that when the number of factors is more than 2, the number of derived cells from the cross-tabulation of the quantiles of all factor scores might be too large, which needs strong subjective decisions to collapse them into mutually exclusive groups (1, 19, 41). In contrast, LCA is well-suited to an issue of identifying the heterogeneity embedded in the sample and classifying the sample into mutually exclusive subgroups. Because LCA is based on the FMM, which postulates that there are subgroups with different dietary structures, and these subgroups should have different food consumption probability distributions (Figure 1) (19, 42, 43). Through FMM, the distribution is heterogeneous across the overall sample but homogeneous within subgroups, which maximize the differences of the dietary patterns derived by LCA in the food consumption probability (44). The characteristics of LCA make it easier to compare the health outcome between the individuals because an individual belongs to only one class and the health outcome is also specific to individuals within each class.

Most previous research on dietary patterns and the breast cancer risk was conducted by the FA method in Western populations. An inverse association with the *Prudent*-dietary pattern and a positive correlation with the *Western*-dietary pattern of the breast cancer risk have been found in most studies (45–47). However, the results were not consistent. Although there were a few studies on dietary patterns and the breast cancer risk in Asian women, conflicting results were also noted (48–52). In this study, based on LCA results, there is no significant difference between breast cancer and the *Prudent-*class, *Western*-class, or *Chinese traditional*-class. What deserves attention is the *Picky*-class, which is similar to the “*Salty*-pattern” in a previous study (53), women in the *Picky*-class were characterized by higher extreme probabilities of non-consumption on specific foods, the highest probabilities in consumption of pickled foods, and the lowest probabilities in consumption of cereals, soy foods, and nuts. The risk of the *Picky*-class may come from an imbalance diet that could lead to the loss of certain vital nutrients and a high consumption of pickled foods that are prone to inflammation (33).

The strength of this study included the study design that allows us to compare the predictability and comparability of biomarkers and the disease risk between the dietary patterns derived from different posterior methods, and this study provides evidence that the dietary patterns derived from posterior methods are biologically meaningful and demonstrates the role of dietary patterns in the disease risk. A understanding of the derivation of dietary patterns will advance the application of dietary patterns in nutrition research. The results of this study indicated that the dietary pattern derived from the FA is suitable for analyzing the synergistic effect of food effects on biomarkers, while the dietary patterns derived from LCA were used to compare the disease risk among people with a different diet structure. The limitation of the study is that both LCA and FA methods are highly data-driven, and a cross-validation with other independent samples in the future is required (54, 55). The next work is to compare the dietary patterns derived by FA and LAC concerning other biomarkers and health outcomes for a better understanding of the utility of these methods in nutritional epidemiology research.

## Conclusion

In conclusion, FA is suitable for an understanding of the correlations between dietary intake and analyzing the synergistic effect of food intake; LCA divides people into mutually exclusive subgroups with different diet structures, which is conducive to compare the disease risk between the groups. We recommend the use of flexible modeling approaches capable of being adapted to specific research.

## Data Availability Statement

The datasets generated during and/or analyzed during the current study are available from the corresponding author on reasonable request.

## Ethics Statement

This study was conducted according to the guidelines laid down in the Declaration of Helsinki and all procedures involving human subjects/patients were approved by the Jiangsu Center for Disease Control and Prevention Ethical Committee. The subjects/patients provided their written informed consent to participate in this study.

## Author Contributions

All authors contributed to the preparation of the manuscript. MW, SC, and PMW: designed and conducted the study. QRZ, ZZ, and JYZ: developed diet indices and data collection. SC and LCL: performed the statistical analyses and drafted the manuscript. PMW and MW: interpreted the data, critically revised the manuscript, and had full responsibility for the analyses and interpretation of the data.

## Funding

This study was supported by World Cancer Research Fund (2011/RFA/473).

## Conflict of Interest

The authors declare that the research was conducted in the absence of any commercial or financial relationships that could be construed as a potential conflict of interest.

## Publisher's Note

All claims expressed in this article are solely those of the authors and do not necessarily represent those of their affiliated organizations, or those of the publisher, the editors and the reviewers. Any product that may be evaluated in this article, or claim that may be made by its manufacturer, is not guaranteed or endorsed by the publisher.
