# Supplementary figures and images for: Association Between Dietary Patterns and Plasma Lipid Biomarker and Female Breast Cancer Risk: Comparison of Latent Class Analysis (LCA) and Factor Analysis (FA)
Source: Front Nutr. 2021 Dec 9;8:645398. doi: 10.3389/fnut.2021.645398 (PMC8698123; doi:10.3389/fnut.2021.645398)

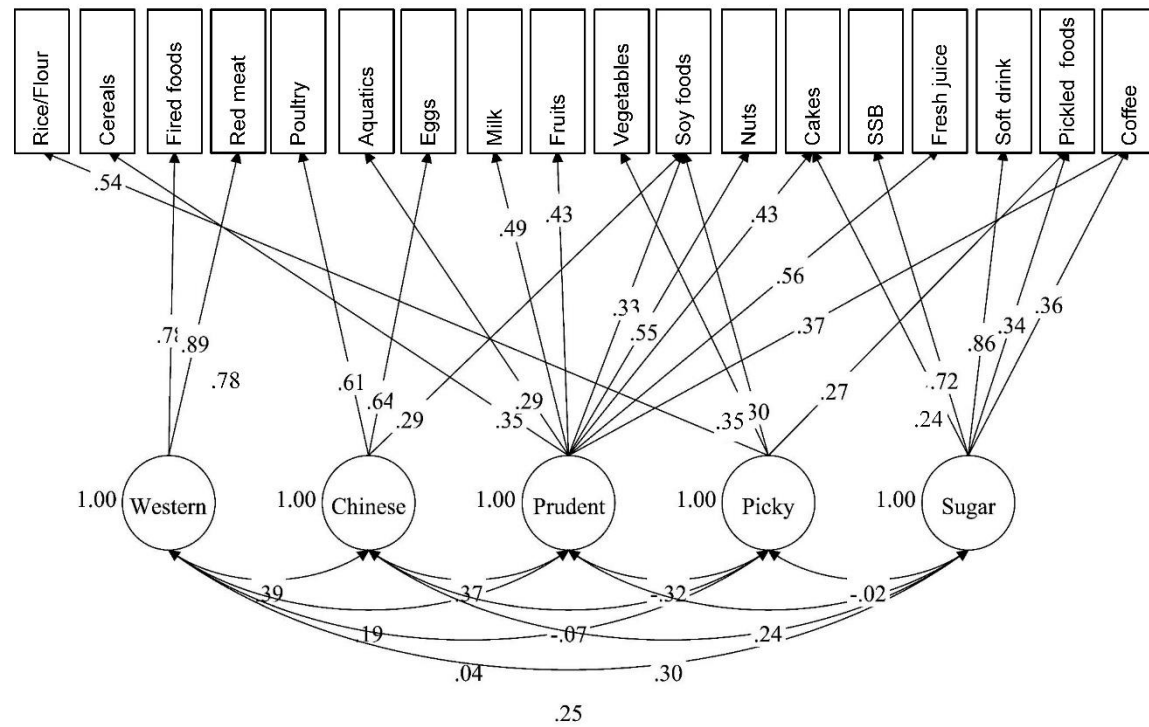

**Figure 1** Confirmatory factor analysis (CFA)

Supplement: Supplementary file 1 [file Image_1.pdf]
